# Books and Pamphlets Received

**Published:** 1888-03

**Authors:** 


### BOOKS AND PAMPHLETS RECEIVED.

*The Physicians' Dose and Symptom Book*, containing the doses and uses of all the principal articles of the Materia Medica and Officinal Preparations, arranged in alphabetical order, and tables of Weights and Measures; Rules to Proportion the Doses of Medicine; Common Abbreviations, etc; Preparation and Modes of Administration; List of Imcompatibles; Hints on Treatment; Table of Symptoms. By Joseph H. Wyeth, M. D., Professor of Histology and Microscopy, Cooper Medical College, San Francisco; Author of the Micoscrope, etc. Seventh edition rewritten and enlarged. Philadelphia: P. Blakiston, Son & Co. 1887.

A very useful book to the practitioner.

*The Curability of Insanity and the Individual Treatment of the Insane.* By John S. Butler, M.D., Hartford, Connecticut. Late Physician and Superintendent of the Connecticut Retreat for the Insane, etc. New York and London. G. P. Putnam's Sons. 1887.

A little work of 59 pages, well and ably written, and elegantly printed.

*Athothis.* A satire on modern medicine. By Thomas C. Minor. Cincinnati. Robert Clarke & Co. 1887.

The work is written in that quaint and humorous style which, while furnishing entertainment to the reader, is made the vehicle of many sharp and critical hits.

